# The role of gut barrier dysfunction in postoperative complications in liver transplantation: pathophysiological and therapeutic considerations

**DOI:** 10.1007/s15010-024-02182-4

**Published:** 2024-02-07

**Authors:** Stelios F. Assimakopoulos, Sanjay Bhagani, Ioanna Aggeletopoulou, Efthymios P. Tsounis, Emmanuel A. Tsochatzis

**Affiliations:** 1 Division of Infectious Diseases, Department of Internal Medicine, Medical School, University of Patras, University Hospital of Patras, Rion, 26504 Patras, Greece; 2 Department of Infectious Diseases/HIV Medicine, Royal Free Hospital, London, UK; 3 Division of Gastroenterology, Department of Internal Medicine, University Hospital of Patras, Patras, Greece; 4 UCL Institute for Liver and Digestive Health, Royal Free Hospital and UCL, London, UK

**Keywords:** Liver transplantation, Intestinal barrier, Intestinal permeability, Endotoxemia, Bacterial translocation, Postoperative infections

## Abstract

**Purpose:**

Gut barrier dysfunction is a pivotal pathophysiological alteration in cirrhosis and end-stage liver disease, which is further aggravated during and after the operational procedures for liver transplantation (LT). In this review, we analyze the multifactorial disruption of all major levels of defense of the gut barrier (biological, mechanical, and immunological) and correlate with clinical implications.

**Methods:**

A narrative review of the literature was performed using PubMed, PubMed Central and Google from inception until November 29th, 2023.

**Results:**

Systemic translocation of indigenous bacteria through this dysfunctional barrier contributes to the early post-LT infectious complications, while endotoxin translocation, through activation of the systemic inflammatory response, is implicated in non-infectious complications including renal dysfunction and graft rejection. Bacterial infections are the main cause of early in-hospital mortality of LT patients and unraveling the pathophysiology of gut barrier failure is of outmost importance.

**Conclusion:**

A pathophysiology-based approach to prophylactic or therapeutic interventions may lead to enhancement of gut barrier function eliminating its detrimental consequences and leading to better outcomes for LT patients.

## Introduction

Liver transplantation (LT) has emerged as an established and well-accepted therapeutic option for patients with acute and chronic liver failure and hepatocellular carcinoma. With advances in surgical techniques and medical management, survival at 1-, 5-, and 10-year post-transplant is about 84%, 72%, and 61%, respectively, according to data from the European Liver Transplantation Registry (ETLR) for 147.392 patients transplanted between 1988 and 2020 [1]. Short-term (in the first 90 days post-transplantation) or in-hospital mortality remain an important problem in LT patients. Although a recent study from the United Kingdom reported an significant improvement in short-term mortality over time, data from several cohorts of LT patients show a relatively high mortality rate (10–15%) [2–4]. Infections represent the main cause of overall mortality in LT, and is also the predominant cause of short-term in-hospital mortality accounting for one third of deaths [2, 3]. Post-liver transplant infections are influenced by immunosuppression, environmental exposure, and surgical complications, while their pattern evolves over time. In the first month post-LT, nosocomial, donor-derived, and surgery-related infections prevail, while with the establishment of immunosuppression, especially in the first 6 months after LT, infections from opportunistic pathogens emerge [5]. With regard to the early postoperative infections in LT, the facts that (i) most of them are caused by gastrointestinal-residing bacteria, (ii) the bacterial translocation process occurs in 26–33% of LT patients up to one month post-LT, and (iii) normalization of patients’ gut microbiota with pre- and probiotics administration reduces the incidence of postoperative bacterial infections, support the possible pathogenetic role of the bacterial translocation process [6–11].

Since gut barrier dysfunction is a pivotal pathophysiological alteration in cirrhosis, it is reasonable to hypothesize that replacing the cirrhotic liver will resolve the dysfunction of the gut–liver axis and restore the gut barrier function. On the other hand, the major operational procedures and time required for replacing the diseased liver, blood loss and multiple transfusions, ischemic time and ischemia/reperfusion (I/R) injury, as well as, immunosuppressive treatments, place the patient in a postoperative critical condition with deterioration of gut barrier function with consequent bacterial and endotoxin translocation leading to infections with significant morbidity and mortality [12–14].

## Methods

A narrative literature search was conducted using PubMed, PubMed Central and Google from inception until November 29th, 2023. Several search terms were used to identify relevant literature: “intestinal barrier”, “gut barrier”, “intestinal permeability”, “gut permeability”, “intestinal barrier dysfunction”, “gut barrier dysfunction”, “intestinal injury”, “bacterial translocation”, “microbial translocation”, “endotoxin translocation”, “endotoxemia”, tight junctions”, “intestinal apoptosis”, “intestinal oxidative stress”, “infections”, “postoperative infections”, “SIRS”, “complications”, “mortality”, “therapy” and “treatment”. combined with the terms “liver transplantation”, “orthotopic liver transplantation”. Results were screened for appropriateness by the first author, according to title and abstract. Most relevant papers were further assessed by full content and their references were also reviewed and assessed when were found relevant. Papers were subsequently organized in subfolders according to planned subsections of this review to retrieve more easily the relevant information. Only English language articles were included. Article types included clinical studies, experimental studies, clinical trials and reviews.

### The gut barrier

The gut barrier consists of three major levels of defense: (i) the biological barrier (gut microbiota), (ii) the mechanical barrier (intestinal epithelial cells and their interconnections), and (iii) the immune barrier (immune cells in gut mucosa and lamina propria and gut-associated lymphoid tissue) [12]. Gut microbiota prevents growth of pathogenic bacteria through antagonism for nutrients and exerting colonization resistance, interacts with epithelial cells supplying them with energy through secretion of diverse metabolites (e.g., short-chain fatty acids (SCFA), such as butyrate, propionate, and acetate) and contributes to harmonic immune regulation through interaction with mucosal immune cells [15]. The intestinal epithelium consists of a single layer of cells tightly sealed to each other through tight junctions (TJ), which restrict the passage of ions, molecules and cells through the paracellular space serving as a critical line of defense [16]. Immune cells within the mucosal tissue further support the defense mechanisms against invading pathogens. Macrophages located beneath the epithelium can phagocytize microbes and release a portfolio of diverse pro- and anti-inflammatory cytokines, whose balance is pivotal for immune regulation [17]. Dendritic cells are responsible for capturing, processing, and presenting microbial antigens to various adaptive immune cells. T lymphocytes act promptly against pathogens by eliminating infected cells, secreting cytokines, and orchestrating immune responses [18]. An additional layer of protection is offered by the gut vascular (endothelial) barrier, which restricts the passage to microbes and their products to the systemic circulation [12].

### Gut barrier dysfunction in patients with end-stage liver disease (ESLD)

**Fig. 1:**
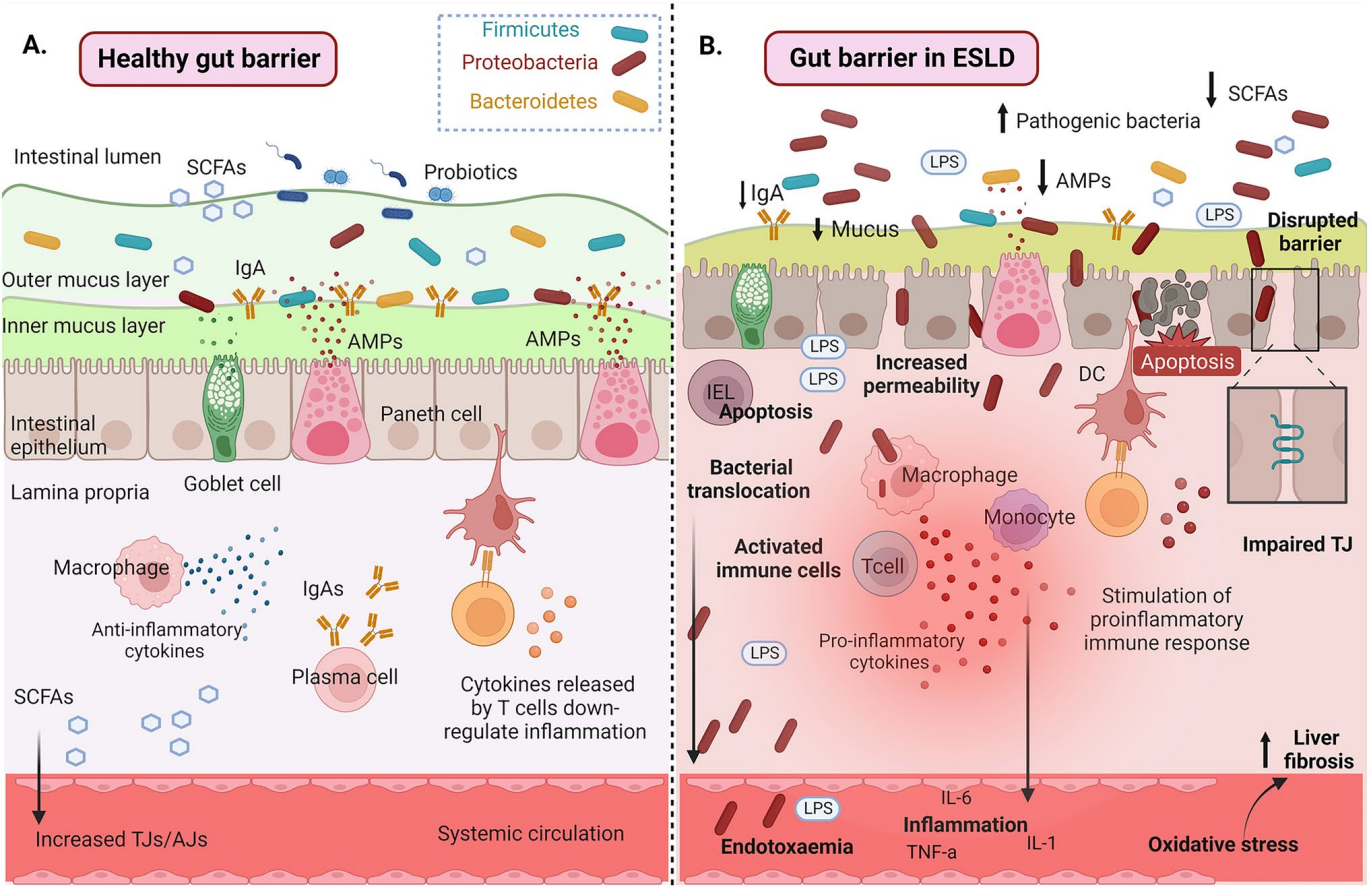
**A** The normal gut barrier function in healthy men and **B** gut barrier dysfunction in ESLD. In ESLD, the integrity of the gut barrier is compromised; the gut microbiota composition is altered (dysbiosis) and characterized by the proliferation of opportunistic pathogenic microorganisms that generate detrimental substances and the reduction of commensal microorganisms who are responsible for producing beneficial metabolites, like short-chain fatty acids. The integrity of the mechanical barrier is disrupted through increased apoptosis/decreased proliferative response of epithelial cells and reduced expression of tight junctions, leading to increased paracellular permeability. The intestinal immune system presents decreased secretion of antimicrobial peptides, dysfunctional response to translocated bacteria with decreased clearance and over-secretion of proinflammatory mediators. These alterations lead to increased bacterial and endotoxin translocation in portal circulation and thereafter, through a dysfunctional liver, to the systemic circulation. Systemic inflammation and oxidative stress contribute to the progression of liver injury and fibrosis and further aggravate gut barrier dysfunction leading to a vicious cycle. This figure was created using BioRender. *SCFAs* short-chain fatty acid, *AMPs* antimicrobial peptides, *IgA* immunoglobulin A, *TJ* tight junctions, *AJ* adherent junctions, *ESLD* end-stage liver disease, *LPS* lipopolysaccharides, *IEL* intraepithelial lymphocyte, *DC* dendritic cell, *IL-6* interleukin 6, *TNF-α* tumor necrosis factor alpha, *ROS* reactive oxygen species, *Th* T-helper, *IFN-γ* interferon γ, *Treg* T-regulatory cell, *ZO-1* zonula occluden-1, *VDR* vitamin D receptor, *RXR* retinoid X receptor, *IECs* intestinal epithelial cells, *NOD2* nucleotide-binding oligomerization domain protein 2, *CAMP* cathelicidin antimicrobial peptide, *TLR* Toll-like receptor, *DEFB2/HBD2* antimicrobial peptide defensin β2, *NF-κB* nuclear factor kappa-light-chain-enhancer of activated B cells, *IgA* immunoglobulin A, *JAM* junctional adhesion molecule

It has been well demonstrated by experimental and clinical studies that liver cirrhosis is associated with intestinal barrier dysfunction and increased gut permeability (Fig. 1) [19–21].

Increased permeability of the intestinal barrier promotes systemic endotoxemia, which subsequently activates a systemic inflammatory response associated with the development of serious complications of cirrhosis from diverse organs [20, 22–27]. According to previous studies by our group, gut barrier dysfunction in liver cirrhosis and increased intestinal permeability are associated with reduced expression of the enterocytes’ TJs molecules occludin and claudin-1 [28]. Other factors implicated in the cirrhosis-induced gut barrier dysfunction decreased degenerative response of the intestinal epithelium, which promotes its vulnerability to injurious insults, and decreased expression of the antimicrobial peptides α-defensins, which represents an important component of gut immunological barrier [29]. All the aforementioned alterations of the intestinal mucosa were more prominent in patients with decompensated liver disease who presented higher levels of systemic endotoxemia and this might be relevant for patients who are eligible for LT.

### Evidence for gut barrier dysfunction in LT patients

**Fig. 2:**
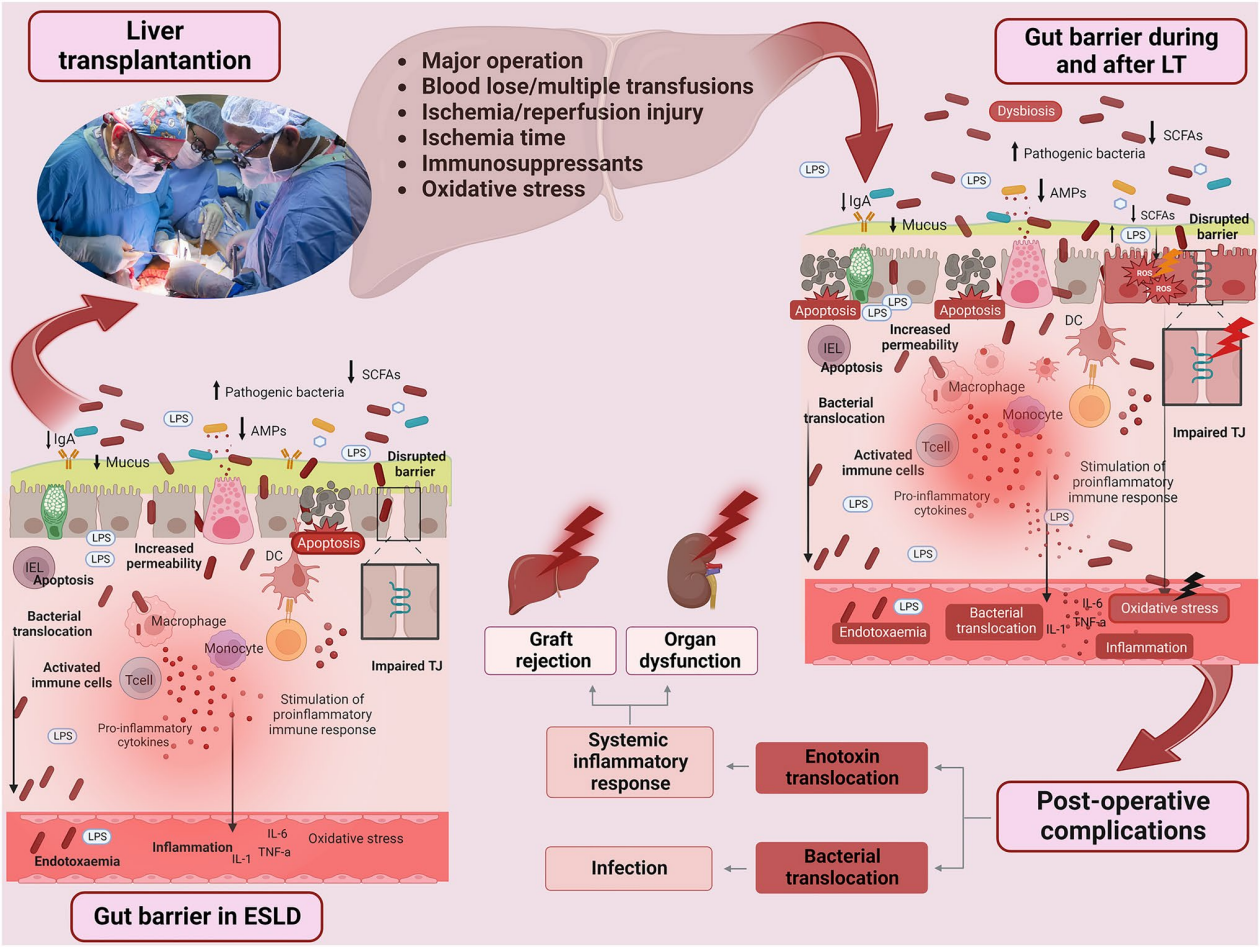
Schematic representation of the association between gut barrier dysfunction and postoperative complications in liver transplantation. Patients with ESLD already present gut barrier dysfunction, which is further aggravated by the complex and long-time operational procedures. Liver transplantation can lead to significant alterations in the gut microbiota due to several factors including antibiotics, immunosuppressants and alterations in immune function. Peri- and post-LT intestinal flora is characterized by decreased bacterial diversity, depletion of beneficial bacteria and overgrowth of pathogenic species. This exaggerated dysbiosis in conjunction with surgical stress, blood loses / multiple transfusions and ischemia/reperfusion injury, which promotes systemic oxidative stress, further injure the gut barrier promoting enterocytes’ apoptosis and TJs disruption, thus increasing gut permeability. The microbiome alterations in conjunction with immunosuppressants lead to subsequent remodeling of the intestinal immune system. The adaptive arm of the intestinal immune system is impaired, characterized by a functional exhaustion of effector B cells leading to deficient production of secretory IgA. Passage of luminal bacteria to the gut submucosa, along with an altered expression of innate immunity receptors, particularly TLR2 and TLR4, trigger various pro-inflammatory downstream pathways. Systemic translocation of indigenous bacteria, through this dysfunctional gut barrier, contribute to the early post-LT infectious complications. At the same time, endotoxin translocation activates the systemic inflammatory response, which is implicated in non-infectious complications, including renal dysfunction and graft rejection. This figure was created using BioRender. *LT* liver transplantation, *SCFAs* short-chain fatty acid, *AMPs* antimicrobial peptides, *ROS* reactive oxygen species, *IgA* immunoglobulin A, *TJ* tight junctions, *ESLD* end-stage liver disease, *LPS* lipopolysaccharides, *IEL* intraepithelial lymphocyte, *DC* dendritic cell, *IL-6* interleukin 6, *TNF-α* tumor necrosis factor alpha

Aggravation of an already dysfunctional intestinal barrier in ESLD patients subjected to LT might be promoted by numerous factors: major operation, I/R injury, loss of blood, multiple transfusions, mechanical ventilation, and immunosuppressive treatments (Fig. 2).

Several preclinical studies with experimental animals subjected to LT have demonstrated increased postoperative bacterial and endotoxin translocation [30]. A previous cross-sectional study with 35 LT patients at diverse times post-transplantation showed that they presented increased intestinal permeability measured by ^51^Cr-EDTA excretion, compared to healthy individuals, and comparable to that observed in cirrhotic patients [31]. In a previous study, with 81 LT patients, plasma endotoxin was significantly increased during operation (at the end of the anhepatic phase), while higher endotoxin levels preoperatively and at the end of the anhepatic period were associated with graft failure and high mortality [13]. Another study with 32 LT patients demonstrated increased portal and systemic endotoxemia up to 120 min after reperfusion. Furthermore, in a prospective longitudinal study with 17 LT patients, which evaluated gut barrier function by measuring peripheral blood endotoxin concentrations in the perioperative period (before LT and postoperative up to day 21), endotoxin concentration was increased postoperatively, both in patients with and without infectious complications, but patients with postoperative infections had significantly higher endotoxin values [32]. Several experimental and clinical studies have demonstrated that liver-related surgery, other than LT, is associated with gut barrier disruption. We have previously demonstrated that experimental animals subjected to partial hepatectomy present significant alterations of the intestinal mucosa with induction of mucosal atrophy, apoptosis and oxidative stress leading to gut barrier dysfunction and endotoxemia [33, 34]. Intestinal injury with increased gut permeability leading to systemic endotoxemia is also evident in patients subjected to liver resection surgery [35, 36]. To the best of our knowledge, there are no experimental or clinical data comparing the magnitude of the hepatectomy-induced gut barrier impairment between the LT context and other conditions. However, preclinical studies have shown that gut barrier disruption is attenuated when a laparoscopic approach was employed rather than an open surgery for major liver resection [37]. Therefore, it is possible that a pure laparoscopic living donor liver transplantation, when performed in appropriate candidates by an experienced transplantation team, might lead to improved patient outcomes through attenuation of gut barrier injury [38]. Also, another experimental study exhibited that radiofrequency-assisted hepatic resection could mitigate the histologic alterations and immune system dysregulation in the intestinal mucosa resulting from the procedure and help maintain gut barrier homeostasis [39].

### The gut mechanical barrier in LT

Most evidence for the mechanisms of intestinal injury in LT originated from preclinical studies with experimental animals. Under electron microscope, the intestinal mucosa of LT rats presented evidence of derangement, manifested by intestinal villus epithelial cell necrosis, loss of ultrastructure, shortened mucosal villi length, increased gap between epithelial cells, accompanied by capillary congestion, interstitial edema, and inflammatory cell infiltration [30]. Widened space between intestinal epithelial cells observed in microscopic studies, which functionally means an opened paracellular route for translocation of luminal endotoxin and microbes, was shown to be based on reduced expression of the TJ proteins occludin and ZO-1 [40]. Intestinal epithelial cell injury has been further demonstrated by increased levels of several biomarkers in serum, including diamine oxidase, intestinal-fatty acid binding protein 2 (I-FABP-2), and d-lactate [40]. Moreover, enterocytes’ apoptosis is induced through activation of the toll-like receptor 4 (TLR4) / nuclear factor kappa B (NF-κB) signaling pathway, which leads to overexpression of proinflammatory cytokines like tumor necrosis factor alpha (TNF-α) and interleukin (IL)-1*β* in the intestinal mucosa [30, 40, 41]. In this proinflammatory milieu, oxidative stress seems to play a pivotal role in the LT-associated intestinal injury, as the mucosal antioxidants superoxide dismutase, glutathione S-transferase α1 (GST*α*1), and glutathione (GSH) were decreased [40]. Proinflammatory mediators and oxidative stress may underlie enterocytes’ TJ disruption, because it has been previously demonstrated that TNF-α overexpression downregulates occludin’s promoter and oxidative stress disrupts the TJ structural complex by modulation of the assembly, localization, expression and function of its molecular components [42, 43]. In addition, increased mucosal oxidative stress might be an important promoter of enterocytes’ apoptosis [43]. Most importantly, a causative role of oxidative stress in LT-associated intestinal injury is supported by the observation that intestinal mucosal changes are significantly ameliorated after activation of the nuclear factor erythroid 2-related factor (Nrf2) / heme oxygenase (HO)-1 signaling pathway that reduces oxidative stress [40].

### The gut biological barrier in LT

**Fig. 3:**
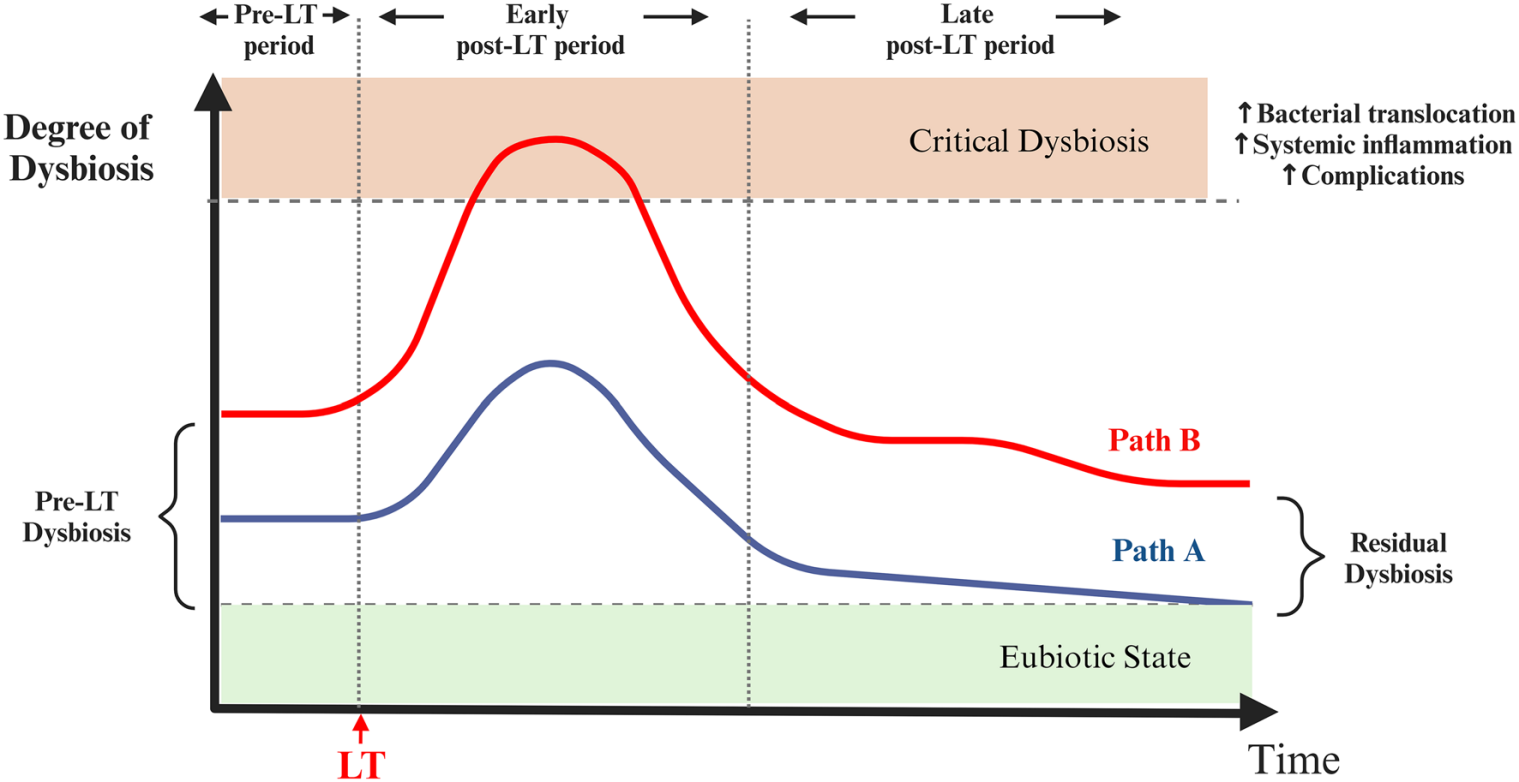
Diagram of gut dysbiosis dynamics following liver transplantation (LT). Critical dysbiosis describes a state of profound imbalance that is particularly associated with critical illness. In the context of critical dysbiosis, the microbiota undergoes significant and impactful changes, potentially leading to complications and adverse health outcomes. The extent of gut microbiome dysbiosis emerges as a major determinant of LT outcomes. Path A represents the subset of patients who sustain a certain degree of gut microbiome homeostasis at the time of LT. These patients could be more resilient to surgical stress, ischemia/reperfusion injury, and immunosuppressive treatment. Following an early post-LT phase marked by dysbiosis deterioration, these individuals gradually restore their intestinal microflora. Path B represents the subset of patients who harbor a microbiome exhibiting poor bacterial diversity, marked by a depletion of protective taxa and colonization by multi-drug-resistant bacteria at baseline. These individuals are at an increased risk of progressing toward an increasingly dysbiotic state associated with aberrant bacterial translocation, excessive endotoxemia, and systemic inflammation. This condition may precipitate detrimental infectious or other immune-mediated complications. A level of “residual” dysbiosis persists even in the late post-LT period, and its magnitude might hold clinical significance. The dynamic interplay between the gut microbiome and the host in the context of liver transplantation holds potential implications for customizing therapeutic strategies and enhancing overall outcomes. *LT* liver transplantation

The dynamics of gut microbiota composition following LT and their influence on disease prognosis are important research areas. Several studies demonstrate that in the peri-operative and early post-LT periods, intestinal microflora undergoes profound alterations with further deterioration of the preexisting dysbiotic state, as illustrated in Fig. 3 [44–46].

Lai et al. [44] analyzed fecal samples from LT recipients during the first and second weeks following transplantation, and their findings revealed a reduction in bacterial diversity accompanied by the loss of numerous signature species, including *Ruminococcus*, *Blautia*, and *Bifidobacterium*. Kato et al. [45] reported that microbial diversity, as assessed by the Shannon diversity index, significantly decreased during the first three weeks after the procedure. In line with these, Wu et al. [46] showed that fecal samples within 6 months from LT were depleted of beneficial bacteria, like *Bifidobacterium*, *Faecalibacterium prausnitzii* (*F. prausnitzii*), and *Lactobacillus*, while pathogenic species such as *Enterococcus* were enriched. Interestingly, these studies suggest that a partial restoration of gut microbiome homeostasis is gradually achieved with the recovery of diversity and proliferation of eubiotic species [45, 46]. Indeed, analysis in stable patients after 6 months from LT revealed a rebound in microbial diversity with a relative predominance of indigenous genera from the *Ruminococcaceae* and *Lachnospiraceae* families and a decrease in potentially harmful *Enterobacteriaceae* genera, including *Escherichia, Salmonella*, and *Shigella* [47]. Amelioration of the intestinal microbiota was accompanied by the recovery of the circulating bile acid pool, a mitigation in endotoxemia, and a restoration in both serum lipidomic and urinary metabolomic profiles [47]. Intriguingly, the increase in *Firmicutes* and the reduction in *Proteobacteria* appeared to correlate with cognitive improvement, demonstrating that gut microbiome changes translate to significant clinical outcomes [47]. In another study by Bajaj et al. [48] it is suggested that while LT leads to a restoration in the intestinal microflora, dysbiosis may persist in certain patients even after 6 months, potentially resulting in unfavorable outcomes. Particularly, a significantly higher relative abundance of *Proteobacteria* and lower levels of *Firmicutes* were observed in post-LT patients who did not experience cognitive and health-related quality of life improvements [48]. In accordance, Lee et al. [49] exhibited that long-term LT patients maintain a certain degree of dysbiosis, characterized by a deprivation of the commensal butyrate-producing *Faecalibacterium* and an increased abundance of the pathogenic *Bacteroides.* Intriguingly, in vitro analysis showed that supplementation with *F. prausnitzii* and butyric acid exerted beneficial immunomodulatory effects by enhancing the Treg/Th17 ratio [49]. Of note, in a recent prospective study by Salimov et al., the levels of *Proteobacteria* were decreased in patients who developed acute graft rejection in comparison to those who remained free of this complication [50]. Reduced microbial diversity, along with an increase in *Proteobacteria* and *Actinobacteria*, as well as a decrease in *Firmicutes*, have all been previously linked to acute cellular rejection in liver transplant recipients [45]. Importantly, Annavajhala et al. [51] report that distinct pre-transplant bacterial signatures could predict post-LT colonization with multi-drug-resistant (MDR) bacteria, while peri-operative loss of microbiome diversity was associated with clinical complications, including bleeding, biliary leak, or biliary stricture. In turn, colonization with MDR bacteria in the pre-operative period was a predictor of poor diversity in the post-LT period. Lastly, clinical indices such as model for end-stage liver disease (MELD) and Child Pugh (CP) scores may reflect changes in the gut microbiome [52].

### The gut immunological barrier in LT

The intestinal immune system and the gut microbiome engage in a mutual and dynamic relationship, influencing each other's development and function. The significance of gut microbiome alterations following liver LT and the subsequent remodeling of the intestinal immune system is an emerging field of research. In post-transplant animal models, persistent endotoxemia and bacterial translocation, along with an altered expression of innate immunity receptors, particularly TLR2 and TLR4, which recognize microbial patterns and trigger various pro-inflammatory downstream pathways, have been observed [53]. TLR-4 downstream signaling is a widely recognized pathway in liver diseases, playing a pivotal role in fibrogenesis and inflammation. Endogenous ligands of TLR4, originating from damaged matrix and injured cells, actively participate in this signaling cascade. Particularly, in the context of LT where tolerance to TLR4 stimuli is compromised, the combined effect of endotoxin translocation and endogenous ligands could exacerbate systemic inflammation and fibrogenesis [54]. Notably, the relative counts of *Bacteroides*, *Lactobacilli*, and *Enterobacteria* were restored in the post-transplant group but not in the liver cirrhosis sub-group [53]. This suggests that the fine-tuning of mucosal immunity may be gradually achieved at a later stage. Human data demonstrate the impairment of the adaptive arm of the intestinal immune system during the post-LT period, marked by a functional exhaustion of effector B cells leading to deficient production of secretory IgA, a crucial component for microbial control on mucosal surfaces [46].

An integral aspect of the post-liver transplantation (LT) period involves the implementation of immunosuppressive treatments, which often entail the long-term administration of medications such as tacrolimus, cyclosporine, mycophenolate mofetil, and prednisone. In animal models, each of these agents induced significant modifications in the fecal microbiome at the family level, while combined treatment catalyzed the elimination of the genus *Clostridium *sensu stricto in ileal samples and promoted the proliferation of uropathogenic *E. coli* strains [55]. In addition, combined immunosuppressive therapy resulted in reduced expression of C-type lectins Reg3β and Reg3γ, which are important for controlling intestinal microbiota, along with downregulation of IL-22 expression in the ileum [55]. The administration of cyclosporine has been associated with impaired priming of the IgA response against cholera toxin [56]. In a different experimental model, the administration of tacrolimus led to a noteworthy modification in the Treg cell population within the colonic mucosa [57]. In human studies, patients with higher tacrolimus level/dose ratios experienced a significant decline in the functional microbiome, as represented by *Faecalibacterium*. In contrast, this decline was reversed in tolerant patients, who no longer required immunosuppression and displayed an increase in Treg cells [49]. In this setting, Bajaj et al. [47] report that although tacrolimus might decrease the Firmicutes/Bacteroidetes ratio, in their post-LT cohort receiving tacrolimus, the ratio was increased instead, implying the vital role of a properly functioning liver in facilitating improvements in gut microbiota. Collectively, impaired immune responses associated with gut dysbiosis which are present in patients with ESLD are further deteriorated during the peri-operative and early postoperative period of LT but could at a later phase gradually return to a state approaching normal.

### Clinical implications of gut barrier dysfunction in LT

#### Infectious complications

Preexisting gut barrier dysfunction in patients with ESLD is further aggravated by the complex and long-time operational procedure and its associated I/R injury. Therefore, the bacterial translocation process is promoted during LT surgery and in the postoperative period, contributing to infectious complications [58]. In a prospective longitudinal study with LT patients, evaluating endotoxin concentrations in the perioperative period, patients with post-LT infectious complications had significantly higher endotoxin levels before LT and at the 14 postoperative day compared with those without complications [32]. These findings point toward the importance of gut barrier failure in the postoperative course of LT patients, especially regarding their predisposition to infections. An important problem with LT recipients is that the short-term postoperative bacterial infections are increasingly caused by multi-drug resistant (MDR) pathogens [6, 59]. The indigenous bacterial flora in this patient population might be populated by MDR bacteria [extended-spectrum beta-lactamase (ESBL)-producing or carbapenem-resistant Enterobacteriaceae (CRE), vancomycin-resistant enterococci (VRE), MDR Pseudomonas aeruginosa, carbapenem-resistant Acinetobacter baumannii and methicillin-resistant *Staphylococcus aureus* (MRSA)], which are associated with higher mortality when causing post-LT infections [59, 60]. Colonization of LT-eligible patients with MDR bacteria is attributed to the presence of diverse risk factors, including prolonged waiting times for LT, leading to unavoidable and often repeated hospitalizations, repeated exposure to antibiotics (often advanced options), prolonged stay in intensive care unit (ICU) and immune suppression [6]. Conversely, the need for prolonged post-LT mechanical ventilation or renal replacement treatment or other underlying diseases may further exacerbate gut barrier dysfunction predisposing to MDR colonization and detrimental outcomes [61]. Pre-LT screening for bowel colonization by MDR bacteria might guide the appropriate selection of prophylactic and therapeutic antibiotics thus improving the outcome of these patients [62].

#### Non-infectious complications

Gut barrier dysfunction in LT has been also associated with liver graft failure and renal dysfunction [13, 14]. In a study with 81 LT patients, plasma endotoxin was measured preoperatively, at the end of the anhepatic phase, and on postoperative days 1, 3, and 7. The presence of high endotoxin levels preoperatively and at the end of the anhepatic period was associated with graft failure and high mortality [13]. Based on the results of another study with 76 LT patients, postoperative endotoxemia (at day 7) was considered to be the principal cause of early postoperative renal dysfunction [14]. From a pathophysiological point of view, the connecting link between the gut barrier dysfunction in LT and contribution to renal injury or graft rejection is an endotoxin-stimulated systemic inflammatory response syndrome (SIRS), characterized by the release of various cytokines and vasoactive mediators, such as IL-1, IL-6, IL-8, IL-10, TNF-α, nitric oxide, and endothelin-1, which can induce circulatory and remote organ dysfunction, partially through promotion of reactive oxygen species formation [63–66]. Previous studies have shown that the balance of pro- and anti-inflammatory cytokines, T-helper (Th)-1, Th-2, Th-17, and T-regulatory (Treg) signature cytokines during LT affect graft function, other organs function and patients’ prognosis [67]. T-cell-mediated rejection (TCMR), typically manifesting in the early postoperative period, is marked by the infiltration of diverse inflammatory cells into the liver graft, including neutrophils, eosinophils, macrophages, and lymphocytes. T cells constitute the majority of infiltrating lymphocytes and demonstrate Th1 polarization, the hallmark of TCMR, which is precipitated by the circulating pool of pro-inflammatory cytokines [68]. However, it is important to note that the liver hosts a unique immune niche distinct from the systemic extrahepatic compartment, showing an immunotolerant phenotype that can be optimized by immunosuppressive therapy. The local activity of chemokines exerts important effects on retaining alloactivated lymphocytes at sites of graft injury, reflecting the distinct gene expression patterns between the periphery and the liver [69]. This emphasizes the critical importance of immunoregulatory networks, which function differentially between these compartments [70].

### Pathophysiology of postoperative non-infectious complications in LT: beyond the “classical” bacterial translocation process

In the LT patient, the development of postoperative infections with cultivation of bacteria of enteric origin is possibly related pathophysiologically with the bacterial translocation process. However, not rarely in clinical practice, LT patients may develop in the postoperative period a clinical and laboratory profile of sepsis without isolation of pathogens in relative cultures. Also, non-infectious complications like deterioration of renal function or liver graft rejection are often developed in the context of SIRS with negative culture results. The lack of identification of bacterial pathogens as causative factors of SIRS is usually attributed to the prophylactic administration of broad-spectrum antibiotics. However, this might only partly explain the complex pathophysiological processes evolved in these patients. The same phenomenon in critically ill surgical patients has been described since the 90 s and was nicely interpreted by professor Deitch and colleagues in 2006 with the “gut-lymph” theory of gut-origin sepsis and multiple-organ dysfunction syndrome (MODS) [71]. Based on data from well-designed experimental models and patients, this theory highlights the role of the mesenteric lymph as a carrier of gut-derived danger-associated molecular patterns (DAMPs) to the systemic circulation [71–73]. More specifically, in a dysfunctional gut barrier, microbes gain access to the intestinal submucosa activating the intestinal immunological system and promoting a proinflammatory response, which further aggravates intestinal injury. Then, DAMPs are released in the mesenteric lymphatics passing subsequently to the systemic circulation, promoting a systemic inflammatory response associated with remote organs’ dysfunction, irrespectively of translocation of intestinal microbes or their products beyond the gut or the mesenteric lymph nodes [12]. In LT patients, both explanations, classical bacterial translocation and the gut-lymph hypothesis of sepsis, may occur depending on the patient; however, this is a theoretical concept which remains to be proved.

### Potential therapeutic and preventive measures

Therapeutic or preventing approaches against bacterial and endotoxin translocation in LT patients can be pathophysiologically categorized in two major groups: (a) interventions aiming to reduce the intraluminal pool of microbes and/or their products with potential for translocation or normalize intestinal flora disturbances (selective decontamination of the digestive tract (SDD), probiotics/prebiotics/symbiotics) and (b) interventions aiming at preventing or restoring intestinal barrier injury (early resuscitation, enteral nutrition, immunonutrition, antioxidants). These axes of therapeutic intervention target the gut barrier dysfunction as the motor of the inflammatory response and aim at breaking the vicious cycle of the continuous gut-derived inflammatory activation which induces injurious effects in diverse organs. However, anti-inflammatory treatments can also modulate the post bacterial translocation inflammatory response systemically or at the organ level with positive results. The decision-making process is complex, requires a thorough understanding of the patient's pathophysiology, and should involve a comprehensive evaluation of risk factors, and patient’s overall health. In many cases, a combination of approaches may be employed to achieve the best outcomes, balancing the need for immune tolerance, infection prevention, and minimizing the side effects associated with immunosuppression.

#### Selective decontamination of the digestive tract (SDD)

SDD consists of the use of oral non-absorbable antibiotics plus a short course (3–4 days) of systemic broad-spectrum antibiotics, with spectrum targeting gram-negative aerobic enteric bacteria and minimal action against commensal anaerobic bacteria. SDD has been consistently shown to reduce infections and ventilator-associated pneumonia (VAP) in ICU patients [74, 75]. SDD in liver transplant patients was introduced as a prophylactic strategy against postoperative infections by Wiesner et al*.* in 1988, with reduction of postoperative infections by 50% [76]. Since then, several observational studies and randomized clinical trials have been conducted with conflicting results [77]. However, a meta-analysis of earlier studies on the efficacy of SDD in liver transplant patients demonstrated its beneficial effects [78]. These conflicting data may be attributed to variations in study design and outcome measures. Differences in the types of antibiotic treatments, the timing of their initiation, and the duration of the therapies across studies could contribute to the discrepancies in results. In a cohort study, Gorensek et al. [79] demonstrated that SDD with norfloxacin and nystatin, initiated upon patient inclusion in the active waiting list until the fourth postoperative week, was well tolerated and highly protective against infections. While a trend toward improved short-term survival was observed in the SSD group, long-term mortality did not significantly differ from the control group. Bion et al. [80] in their randomized trial showed that DDS with tobramycin, amphotericin, and polymyxin B prevented colonization of the respiratory tract with gram-negative bacteria but did not reduce systemic endotoxemia in liver transplant recipients. In a randomized controlled trial (RCT) by Arnow et al. [81] infection rates were reduced in patients receiving SDD with gentamicin, polymyxin, and nystatin for more than 3 days before transplantation compared to control patients. However, another RCT using a similar antibiotic combination (with over 85% receiving treatment more than 3 days before the operation) through day 21 after LT failed to demonstrate improvement in infection and mortality rates [82]. In a placebo-controlled trial including 58 patients Zwaveling et al. [83] found no significant protective effects against bacterial infections with SSD with norfloxacin and tablets containing colistin, tobramycin, and amphotericin B. Remarkably, the microorganisms causing infection varied; the SSD-treated group exhibited a notable reduction in infections from Gram-negative bacilli and yeasts, while infections from gram-positive cocci were more evident. In a prospective Spanish cohort, including 1010 LT recipients, SSD with fluoroquinolones for a minimum of 7 days did not confer any benefits in the incidence of early bacterial infections [84]. The risk of selection of MDR microorganisms has raised important concerns about the routine application of SDD in LT patients. In this setting, rifaximin administration in severely ill patients with hepatic encephalopathy was associated with reduced risk of bacterial infections without an increased risk for MDR infections [85].

#### Probiotics, prebiotics, and synbiotics

Probiotics are living non-pathogenic microorganisms, which when administered in optimum amounts promote a healthy gut microbiome with health benefits; prebiotics are specific plant fibers that promote the growth of beneficial bacteria; and synbiotics are a combination of the two [86]. In a recent systematic review and meta-analysis of 12 relevant studies on peri-operative administration of pro-/syn-biotics in liver surgery, including 5 studies in LT, a significant reduction of postoperative infection rate with this strategy was shown [11]. In another recent meta-analysis on the safety and efficacy of combined use of prebiotics and probiotics (Lactobacillus and Bifidobacterium) in patients undergoing LT, it was shown that this intervention leads to lower infections and shorter hospital stay or antibiotic therapy, when compared with conventional nutrition [87]. However, the diversity of the probiotic and prebiotic preparations and administration timeframes used among the studies, point toward the necessity of evaluation of standardized study protocols. With regards to safety of probiotic administration, protocols must be thoroughly evaluated, because prophylactic probiotic administration in ESLD patients with increased gut permeability may lead to probiotic strains translocation with harmful consequences.

#### Timely and careful hemodynamic resuscitation

Gut hypoperfusion represents a pivotal initiative event leading to intestinal injury and gut barrier dysfunction in the critically ill patient. Inadequacy of blood supply to the intestine promote several injurious effects in intestinal epithelial cells, including increased apoptosis, decreased proliferative response and loss of tight junctions’ integrity [88]. These changes are further aggravated during reperfusion (I/R injury) through oxidative stress-mediated mechanisms [89, 90]. Anti-oxidant volume resuscitative therapies have shown promising results regarding prevention of I/R injury [91, 92]. Timely resuscitation to maintain the intravascular volume and cardiac supply is a critical therapeutic manipulation. Balanced fluid administration and early vasopressor use might prevent mucosal edema and beneficially affect gut barrier function, on the contrary with aggressive fluid replacement [93].

#### Enteral feeding

Preservation of the normal structure and function of the gut requires nutritional support. Deprivation of the digestive tract from food nutrients and their associated digestive secretions induces mucosal atrophy and gut barrier dysfunction thus promoting bacterial translocation [94]. In ICU critically ill patients, enteral feeding as compared to total parenteral nutrition was associated with reduced rates of infectious complications and mortality [95–97]. Meta-analyses of clinical trials of enteral nutrition in patients undergoing liver transplantation have shown a positive impact with regard to postoperative infections and length of ICU stay [98]. Early (during 12–24 h) oral feeding is recommended in the current “enhanced recovery after surgery” (ERAS) guidelines for liver resection, liver transplantation, and pancreatoduodenectomy [99].

#### Immunonutrition

The term “immunonutrition” refers to the administration of pharmacologically active nutrients that modulate the metabolic and inflammatory response to surgery or critical illness and enhance immune function. The enteral administration of these substrates in conjunction with the basic nutritional supply is referred to as enteral immunonutrition. Enteral immunonutrition is considered a strategy of direct nutritional support of enterocytes and has been used for prevention of gut barrier injury. The most well studied immunonutrients are glutamine, arginine, ω-3 fatty acids, γ-linoleic acid, and nucleotides [100]. In a meta-analysis of 7 randomized controlled trials involving 501 LT patients, peri-operative immunonutrition significantly reduced the risk of infectious complications and shortened the postoperative hospital stay [101]. However, evidence is not yet considered sufficient for a specific recommendation in ERAS guidelines [99].

#### Antioxidants

High levels of transplanted organ’s oxidative stress, attributed to I/R injury, are a central pathophysiological event in solid organ transplantation. Oxidative stress in LT affects the gut–liver axis and is also systemically spread. Therefore, the trial of antioxidants is a reasonable intervention to prevent organ dysfunction in this setting. Diverse antioxidants including N-acetyl-cysteine and ascorbic acid have been tested with positive results in LT patients in an attempt to prevent liver graft injury [102, 103]. However, there are very limited data regarding the effect of antioxidant treatments on LT-induced gut barrier oxidative injury.

### Future perspectives: is there a role for fecal microbiota transplantation (FMT)?

FMT is currently an established treatment for recurrent Clostridioides difficile infection, while it has been successfully used in several other intestinal and extra-intestinal diseases characterized by intestinal dysbiosis [104]. We have recently shown in an animal model of polymicrobial sepsis that FMT induces a multifactorial improvement of the gut mechanical and immunological barriers, preventing endotoxemia and leading to improved survival [105]. The concept of using FMT to reverse intestinal dysbiosis and improve gut barrier function, potentially leading to attenuated septic complications, has not been previously tested in LT experimentally or clinically. However, FMT for the treatment of recurring CDI episodes and has been successfully used in LT patients [106]. Recent data indicate that FMT can be safely administered to immunocompromised patients, demonstrating comparable safety profiles to immunocompetent individuals [107]. In addition, FMT could exert beneficial immunomodulatory effects that could improve LT outcomes through the replenishment of commensal bacteria and their metabolites. We think that there is a theoretical basis for the study of FMT as a potential therapeutic approach for reversal of gut microbiota alterations and prevention of the gut-derived bacterial translocation and its associated infectious and non-infectious complications in LT recipients.

## Conclusions

LT is associated with a multifactorial disruption of the integrity of the intestinal biological, immunological, and mechanical barrier, promoting the phenomenon of bacterial translocation which is a crucial determinant of early postoperative infections. LT patients are exposed to multiple risk factors for colonization by MDR organisms, which through the bacterial translocation process are implicated in postoperative infections leading to higher morbidity and mortality rates. In addition, endotoxin translocation in the systemic circulation activates a systemic inflammatory response which is implicated in non-infectious complications including renal dysfunction and graft rejection. Beyond advances in surgical techniques and immunosuppressant regimens, emerging pathophysiologically based pharmacological approaches aiming at the restoration of the gut barrier are needed. Physicians handling LT patients should not neglect this important parameter, which might improve the prognosis of the LT patient in terms of morbidity and early in-hospital mortality.
